# Patient perspectives on diagnostic uncertainty and novel diagnostic tests in neuro-oncology

**DOI:** 10.1093/nop/npag021

**Published:** 2026-03-07

**Authors:** Veerle J Ruijters, Marloes M Bauer, Nelleke Tolboom, Tatjana Seute, Leonie N C Visser, Tom J Snijders

**Affiliations:** Department of Radiology and Nuclear Medicine, UMC Utrecht, Utrecht, The Netherlands; Department of Neurology, UMC Utrecht Brain Center, UMC Utrecht, Utrecht, The Netherlands; Department of Radiology and Nuclear Medicine, UMC Utrecht, Utrecht, The Netherlands; Department of Neurology, UMC Utrecht Brain Center, UMC Utrecht, Utrecht, The Netherlands; Julius Center, Department of Bioethics and Health Humanities, UMC Utrecht, Utrecht, The Netherlands; Department of Medical Psychology, Amsterdam UMC, Amsterdam Public Health Research Institute, Amsterdam, The Netherlands; Department of Neurology, UMC Utrecht Brain Center, UMC Utrecht, Utrecht, The Netherlands

**Keywords:** diagnostic tests, diagnostic uncertainty, glioma, patient perspectives, quality of life

## Abstract

**Background:**

In the follow-up of glioma patients, differentiating treatment-associated changes from tumor progression on MRI is challenging. In the Netherlands, this diagnostic uncertainty often leads to watchful waiting, delayed treatment, and patient distress. So far, research on new diagnostic tests has primarily focused on accuracy, while patient perspectives remain largely unexplored. This qualitative study aimed to explore glioma patients’ perspectives on diagnostic uncertainty and the implementation of tests aimed at reducing uncertainty.

**Methods:**

Semi-structured interviews were conducted with 10 diffuse glioma patients (31-70 years old; 50% female) treated at UMC Utrecht, the Netherlands. Two independent coders analyzed interview transcripts thematically to identify key themes in patient perspectives.

**Results:**

Four themes were found: (1) Most patients experienced diagnostic uncertainty, which was stressful for themselves and their loved ones. (2) A new test was considered meaningful if it provided early certainty. Patients wanted certainty to guide treatment, and even without therapeutic options, a certain diagnosis could optimize quality of life by living more consciously. (3) All patients wished to participate in decision-making regarding undergoing new tests, with variable preferences regarding the amount of information to be received. (4) Patients expressed the need for continuity of care, honest communication, and immediate disclosure of test results.

**Conclusion:**

Patients value faster certainty from new diagnostic tests, both to guide treatment for improved survival and quality of life and to optimize quality of life when no therapeutic options remain. Integrating this emphasis on quality of life into diagnostic research may stimulate patient-centered test implementation, ultimately improving neuro-oncological care.

Key Points

- Patients value early diagnostic certainty to guide treatment and optimize quality of life.
- Faster certainty also supports maintaining quality of life when no options remain.
- Patients prefer shared decision-making regarding the diagnostic testing strategy.

Importance of the Study

This qualitative study focused on the perspectives of glioma patients on new diagnostic tests. Traditionally, research of new tests focuses on added diagnostic accuracy, which may vary between tests and patients. Yet, due to the distress experienced by glioma patients and their loved ones from diagnostic uncertainty, they consider *earlier* certainty to be most important. Early certainty could provide reassurance, better treatment options, or guidance in life decisions, improving quality of life. Patients prefer to have a say in the potential use of diagnostic tests. Honest communication—preferably with a consistent care team—and prompt disclosure of results were considered important. Emphasizing patients’ perspective and quality of life in future diagnostic test research—even when tests do not directly influence treatment decisions—may support patient-centered test implementation and improve neuro-oncological care. Our findings challenge the general medical view that diagnostic tests should only be performed if they have therapeutic consequences.

During MRI follow-up of irradiated glioma patients, distinguishing between treatment-associated changes and tumor progression can be challenging.^1^^,^^2^ In the Netherlands, when this diagnostic uncertainty arises, a multidisciplinary team typically recommends watchful waiting with follow-up MRI scans 6 to 12 weeks later for a definitive diagnosis. This can not only delay definitive diagnosis and treatment but also cause prolonged uncertainty and anxiety for patients and their loved ones.^3^^,^^4^ As this diagnostic uncertainty between treatment-associated changes and tumor progression is common, many diagnostic studies in neuro-oncology focus on the accuracy of new tests aimed at distinguishing these entities. Additionally, disease-centered outcomes such as survival and progression-free survival are also seen as ultimate proof.^5^^,^^6^

This focus on diagnostic accuracy and disease-centered outcomes in diagnostic test research contrasts the shift in focus observed in neuro-oncology therapeutic research in recent decades.^7^^,^^8^ Increasing attention has been given to the experience of patients with the disease, incorporating patient-reported outcomes (PROs) in clinical trials for new treatments. Outcomes such as symptoms and health-related quality of life (HRQOL) are now assessed alongside established clinical measures such as survival and progression-free survival.^9^^,^^10^ These PROs are primarily integrated into therapeutic trials in neuro-oncology and other medical fields.^9^ However, there is little emphasis on patient-centered outcomes in neuro-oncology diagnostic test research.

High accuracy is a necessity in diagnostic test research, and although disease-centered outcomes are of importance, patient perspectives must equally be considered in the development and eventual implementation of diagnostic tests, as they are the ones that will experience the impact of the tests. Although some existing literature in neurology and neuro-oncology addresses patient perspectives on already implemented diagnostic tests, research on patient views regarding the implementation of new diagnostic tests remains scarce and is nonexistent in the neuro-oncology field.^11–14^

Although the GRADE methodology for diagnostic tests incorporates patient values and preferences in its recommendations,^15^ neuro-oncological guidelines primarily discuss the implementation of diagnostic tests for distinguishing between treatment-associated changes and tumor progression (such as MRI and PET) in terms of diagnostic accuracy.^16^

Given the inevitable recurrence of the disease, glioma patients will, at some point, face diagnostic uncertainty and experience the impact of these new diagnostic tests. Thus, it is essential to identify their perspectives on diagnostic uncertainty and the implementation of tests aimed at reducing this uncertainty. Their perspectives may then (1) be integrated in further quantitative research on diagnostic test strategies (with patient-centered outcomes) and (2) help to better align implementation of these tests with what truly matters to patients, ultimately improving neuro-oncological care. Therefore, the aim of this qualitative interview study was to explore glioma patients’ perspectives on diagnostic uncertainty and the implementation of tests aimed at reducing this uncertainty.

## Materials and Methods

### Design and Setting

For this qualitative exploratory study, interviews were performed with adult patients diagnosed with diffuse glioma (WHO grades 2-4) in the neuro-oncological treatment center of the UMC Utrecht. Interviews were conducted between May 2025 and August 2025. Written informed consent was obtained from all participants prior to their participation. Research ethics approval was obtained from the local research ethics board. This study is reported according to the consolidated criteria for reporting qualitative research (COREQ).^17^

### Patient Recruitment and Sample Size Justification

Patients were approached from February to July 2025 for participation in the study via the outpatient clinic and/or the local neuro-oncology patient advisory group (consisting of patients with a variety of glioma types), with use of purposive sampling. From these sources, the aim was to obtain a diverse and representative sample of glioma patients, including individuals with different glioma types, genders, and ages. Patients were eligible to participate in the study if they (1) were ≥18 years old, (2) were diagnosed with diffuse glioma (WHO grades 2-4), (3) were at least one month post-surgery, (4) had undergone radiotherapy, and (5) were treated in the neuro-oncological center of the UMC Utrecht. Patients were not eligible if they (1) suffered from severe cognitive impairment and/or mixed aphasic disorders preventing an interview, as determined by their physician, (2) suffered from any other condition, including severe mood or other psychiatric disorders, that (in the investigator’s opinion) would prevent adequate participation in the study, or (3) had an insufficient proficiency in the Dutch or English language.

A sample size of *n* = 10 patients was proposed initially, estimated to hold sufficient information power in relation to our study aims and methods, according to the model of information power in qualitative interview studies by Malterud et al.^18^ Only with regard to the item “use of theory” in their model, and our lack of a strong theoretical base, we considered a larger sample might be warranted. Therefore, we conducted a near-end-of-study evaluation to determine whether data saturation had been achieved, so that adjustments to the sample size could be made if required. When the last 2 interviews yielded no substantial new responses or information (ie, new codes or subcodes added to the coding tree), data saturation was considered complete.

### Interviews

Semi-structured interviews were conducted in a single session and took place in person at the UMC Utrecht, through video calling, or by phone, based on the patient’s preference. The interviews were performed by a neuro-oncology researcher-physician who was not involved in the patients’ care. Glioma patients from the UMC Utrecht Neuro-oncology advisory group have contributed to the development of the semi-structured interviews. The study team drafted the initial version, and one patient subsequently assisted in optimizing the key interview themes and refining the questions for clarity. Two test interviews were conducted with 2 other volunteer patients and their feedback was incorporated leading to the definitive interview structure. This interview guide covered the following topics: experiences with diagnostic uncertainty, perspectives on the implementation of new diagnostic tests, preferred communication (including the amount and detail of information), and preferred role in clinical decision-making regarding the use and frequency of diagnostic tests. For the last topic, the Control Preference Scale was used. This scale measures the degree to which patients wish to take an active, shared, or passive role in decisions regarding their care, ranging from fully autonomous decision-making to entrusting it entirely to the clinician.^19^ Patients were encouraged to freely express any additional needs they felt would be beneficial throughout their care.

Additionally, the following information was obtained from the patient’s medical record and from the interviews: age, gender, ethnical background, educational level, religion, family situation and caregivers, glioma type and grade, date of diagnosis, and treatment. The participants received the written interview introduction and interview questions prior to the interview. The interviews lasted 45-60 minutes. In most cases, participants were interviewed individually; however, in some instances, both the patient and their partner were present. The interviews were audio-recorded and transcribed verbatim. Transcripts were used for analysis, and the audio recordings were deleted once transcription was completed.

### Identification of Themes

Once all the transcripts were completed, thematic analysis was performed with the use of Nvivo 15 software.^20^ The Braun and Clarke 6-step framework was used to guide the thematic analysis process.^21^ A combined deductive and inductive approach was used to categorize the data into codes and sub-codes. The deductive part consisted of identifying and categorizing relevant segments into the following broad predefined categories: experiences with diagnostic uncertainty, perspectives on implementing new diagnostic tests, and preferences regarding communication, such as patients’ information needs and their preferred role in clinical decision-making regarding the use of new diagnostic tests. During inductive coding, codes and sub-codes were developed in a bottom-up manner, emerging directly from the data. Two researchers (V.J.R. and M.M.B.) performed the coding independently. Discrepancies in coding between the 2 researchers were resolved in consensus meetings between V.J.R. and M.M.B., with L.N.C.V. as a third party if needed. As more interviews were coded, the resulting coding tree became more and more refined (V.J.R. and M.M.B.). In the next step, overarching themes were formulated by V.J.R. and M.M.B. and reviewed and refined by the total research team (V.J.R., M.M.B., N.T., T.S., T.J.S., and L.N.C.V.). The research team consisted of researchers and clinicians from different fields (neuro-oncology, nuclear medicine, psychology) and an experienced qualitative researcher.

## Results

Ten patients diagnosed with diffuse glioma, including 3 glioblastomas, at UMC Utrecht were interviewed. All patients had received radiotherapy as part of their treatment and were of Dutch ethnicity. The patient characteristics are given in Table 1.

**Table 1.** Patient characteristics.

No.	Age	Gender	Diagnosis	Grade	Therapy	Native language	Level of education^a^	Religion	Marital status, children
1	68	M	Diffuse glioma	3	Resection Radiotherapy Chemotherapy	Dutch	7	—	Unmarried partnership, divorced, children
2	54	F	Astrocytoma	4	Chemoradiation	Dutch	7	—	Married, children
3	58	M	Glioblastoma	4	Chemoradiation Study treatment	Dutch	6	—	Married, children
4	63	F	Astrocytoma	2	Radiotherapy	Dutch	4	—	Married, children
5	44	F	Oligodendroglioma	3	Resection Radiotherapy Chemotherapy	Dutch	6	—	Divorced, child
6	70	F	Glioblastoma	4	Resection Chemoradiation	Dutch	7	Christian	Married, children
7	56	M	Oligodendroglioma	3	Resection Re-resection Radiotherapy Chemotherapy	Dutch	4	—	Married, children
8	41	M	Oligodendroglioma	2	Resection Radiotherapy Chemotherapy	Dutch	7	Christian	Unmarried partnership
9	56	F	Glioblastoma	4	Resection Chemoradiation Re-resection	Dutch	6	Christian	Unmarried partnership, divorced, children
10	31	M	Astrocytoma	2	Resection Radiotherapy Chemotherapy	Dutch	7	—	Marrried

a The Dutch Verhage scale was used for the classification of educational level (1 = did not complete primary school, 7 = university).^22^

### Thematic Analysis

**Figure 1.**
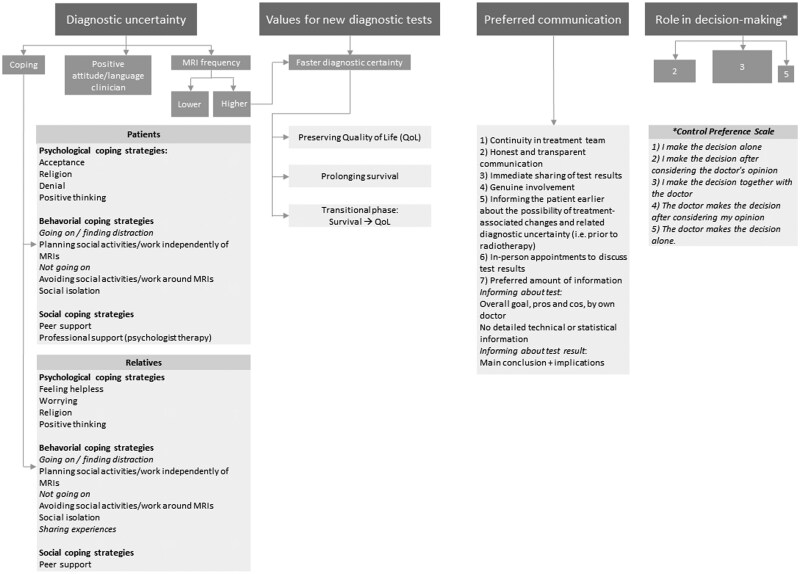
The complete thematic network.

Thematic analysis revealed 4 overarching themes, which will be described below. The complete coding tree is displayed in Figure 1.

#### Theme 1: Most patients experienced diagnostic uncertainty and perceived this as stressful for themselves and their loved ones

The majority of patients had experienced a period (or periods) of diagnostic doubt, that is uncertainty about whether changes seen on MRI were due to treatment-associated changes or tumor progression. All reported this period as stressful, not only for themselves but often also for their loved ones.

In a few interviews, there were indications that a positive attitude and/or certain language from the treating physician influenced the severity of uncertainty experienced by patients, as illustrated by the following quote:

*The doctor said: “I just consulted with the radiologists, and we agree that…” So, with little doubt. Honestly, the strength of the neurologist is also, at some point, being able to take a clear position when necessary. And that’s reassuring, because as a patient, you can’t do that yourself*. (Female patient, 70 years old)

Not only was the period with diagnostic uncertainty stressful, but some patients also reported a certain level of heightened stress throughout the entire follow-up period of the glioma, due to the inevitable recurrence of the disease.

Many patients reported a gradual decrease in these lingering stress levels over time: “*It feels like living with the sword of Damocles hanging above you, suspended by a very thin thread. Over time, that thread becomes thicker and eventually turns into small chains, which then evolve into even heavier links”* (Male patient, 58 years old).

Over time, patients also described to experience an increase in trust when MRI scans remained consistently stable. However, they also reported that this sense of trust and reassurance could easily be disrupted by an uncertain MRI or an MRI showing signs of tumor growth. Nearly all patients experienced varying levels of short-term “anticipation” stress just before receiving the results of a new MRI.

Patients used different types of coping to deal with the distress resulting from diagnostic uncertainty surrounding testing and the long-term uncertainty of the inevitable recurrence. Figure 1 illustrates the various coping strategies reported by patients and their loved ones.

From a psychological point of view, half of the patients reported being able to accept the situation, and tolerate the uncertainty, at least to a certain, yet varying extent. By accepting the situation and realizing that it was out of their control, they were able to let go. In contrast, one patient did not accept the situation and coped with the uncertainty through denial:

*It’s not going to happen to me. It’s not going to win—never. I will survive for 30 more years so I can be there at my youngest child’s wedding and see the birth of my grandchild. That’s how it’s going to be*. (Female patient, 56 years old)

From a behavioral point of view, many patients continued to plan their lives as they would have without the disease. They describe their way of coping as “*going on*,” or as “*finding distraction*,” or both. They deliberately chose not to let MRI appointments influence the planning of, for example, holidays or gatherings with friends and family. “*Yes, life goes on for everyone. So what should we do—sit in a corner, hold each other, and cry? No, that’s not how it works for us*” (Male patient, 56 years old).

Only a few patients put their lives on hold during periods of uncertainty in their treatment and follow-up trajectory. They avoided scheduling social activities or work shifts around MRI appointments, with significant social impact. Several patients who coped through acceptance or by “going on” reported that their relatives had more difficulty dealing with the situation, experiencing greater feelings of helplessness and worry than the patients themselves.

#### Theme 2: A new diagnostic test was considered most meaningful if it provides faster certainty, for reassurance, to guide treatment and thereby improve survival and quality of life, and/or to optimize quality of life by living more consciously when no therapeutic options remained

All participants reported that the implementation of diagnostic tests designed to more accurately distinguish treatment-associated changes from tumor progression would be valuable, when those lead to faster diagnostic certainty. For all patients, the underlying reason for seeking faster diagnostic certainty—apart from reassurance—was the opportunity to initiate the right treatment sooner. The fundamental existential motivations behind this desire include maintaining quality of life, prolonging survival, or a combination of both. This exists on a continuum, with some patients prioritizing maintaining quality of life as their primary goal. *“If I get a treatment which decreases my quality of life, I won’t do it”* (Male patient, 58 years old). While other patients reported to prioritize extending life, even if it means temporarily accepting a decline in quality of life. “*I remember the chemotherapy—oh, how nauseous I was, it was awful. But if it extends my life: absolutely yes. I find life far too beautiful. I enjoy everyone too much, especially my family. I do everything for my children and grandchildren, everything”* (Female patient, 56 years old).

In addition to the desire to initiate the right treatment sooner, some emphasized the importance of knowing the diagnosis of tumor progression even without therapeutic actionability, allowing them to intentionally live and plan their life to the fullest. *“Then I could live a bit more intensely. I might say, ‘I’ll quickly plan a vacation now.’ I know it would be more expensive and require more arrangements, but then I want to do it now. … Then I feel like I still have some control over how things go”* (Male patient, 58 years old).

Beyond prioritizing new diagnostic tests for providing certainty faster, many participants expressed a similar motivation for desiring (more) frequent MRI scans. However, some participants also considered the financial costs and the burden on the healthcare system when expressing their preferences for the frequency of standard MRI scans. “*That is one of the reasons why, at some point, I said, ‘I don’t need an MRI scan four times a year anymore.’ The costs are, of course, skyrocketing, and if it is not really necessary for me, I feel it would be a waste”* (Female patient, 54 years old).

#### Theme 3: All patients wished to participate in decision-making regarding the use of new tests

All patients want to make decisions about the use of a new diagnostic test together with their doctor, with their role ranging from taking the lead to truly sharing the decision. This preference for shared decision-making is consistent among the patients, mostly regardless of the impact of the clinical decision. “*Yes, it’s really together. Like I said, in the end I make the decision myself, but the way we arrive at that decision is definitely a collaborative process. Afterwards, I think I would regret it if I felt like. I shouldn’t have done something, or that I should have. And that has an impact on my quality of life”* (Male patient, 58 years old).

However, a few patients indicated that for choosing the MRI frequency during follow-up—they are comfortable with the doctor making the decision alone. “*Then I follow the doctor’s advice. There have been times when I really thought: I just want MRIs less often. But still, I follow the doctor’s advice, because I believe it’s not for nothing”* (Male patient, 41 years old).

#### Theme 4: Patients emphasized continuity of care, honest communication, and immediate disclosure of test results, with little interest in technical or statistical details

Patients consistently valued honest communication and the immediate sharing of diagnostic test results—such as routine MRI scans—not only during periods of diagnostic uncertainty but throughout the entire treatment trajectory. They highly appreciated receiving the diagnostic test results on the same day as the test and hearing the results immediately upon entering the doctor’s room.

*Before I hit the chair, I want to know the result.* (Female patient, 44 years old)

Most patients also emphasized the importance of continuity in care, specifically seeing the same doctor and nurse practitioner over time, in order to build a trusting relationship.

See Table 2 for the complete overview of patients’ preferred communication from the treatment team, not only during periods of diagnostic uncertainty but also throughout the entire treatment trajectory.

**Table 2.** Overview of patients’ preferred communication from the treatment team.

Honest and transparent communication Rapid sharing of test results^a^ Continuity in care: seeing the same doctor and nurse practitioner Demonstrating genuine involvement Informing the patient earlier about the possibility of treatment-associated changes and related diagnostic uncertainty (eg, prior to radiotherapy) In-person appointments to discuss test results Preferred amount of information^b^ *Informing about test:* Overall goal and pros and cons by own doctor No detailed technical or statistical information *Informing about test result*: Main conclusion and implications

a Receiving the test results on the same day as the test and hearing the results immediately upon entering the doctor’s room.

b Patients varied in how they defined the desired amount of information about diagnostic tests and clinical decisions; what some perceived as concise, others regarded as extensive.

Patients varied in how they defined the desired amount of information about diagnostic tests and clinical decisions. What some perceived as concise, others regarded as extensive. When introducing a new diagnostic test, most patients indicated that they would like to hear the overall goal and a summary of the advantages and disadvantages of a diagnostic test from their own doctor. None of the patients expressed a need for detailed technical or statistical information about diagnostic tests. They also valued hearing the main conclusion of the test result and its implications. The majority of patients would like to see the MRI images during follow-up consultations. Some patients reported that being able to ask questions and hear the doctor’s and multidisciplinary tumor board’s considerations gives them a sense of control. *“I want to understand the doctors’ reasoning for proposing a diagnostic test—what, where, and why? In a situation where I usually have little control, I do feel a sense of agency when they fully involve me in the decision-making process”* (Female patient, 44 years old).

Within the same individual, the desired amount of information may vary across different phases of the treatment process.

*For a long time, I didn’t want to see any scans, and that had a lot to do with uncertainty. I know myself—if the doctor shows me something along with an earlier scan, I’d focus on some little line and think: why isn’t that on the previous scan? But a few scans ago, I thought: okay, go ahead and show me* (Male patient, 41 years old).

One patient shared: *“Before the second surgery, I said: I know what you’re going to say, but I don’t want to hear it. Just tell my husband. And then I think: why does it have to be this way? Why am I not allowed to choose not to hear specific information?”* (Female patient, 56 years old).

## Discussion

The aim of this qualitative interview study was to explore glioma patients’ perspectives on diagnostic uncertainty related to treatment-associated changes and tumor progression, as well as on the implementation of tests designed to reduce this uncertainty. Four main themes emerged from the data: (1) Diagnostic uncertainty is stressful, and (2) obtaining faster certainty through new tests was most important to patients. They wanted certainty not only to guide treatment decisions aimed at improving survival and quality of life but also—even in the absence of therapeutic options—to preserve quality of life by living more consciously. (3) All patients expressed a desire to participate in decision-making regarding the use of these tests, yet their preferences varied in terms of how much information they wished to receive. (4) Patients emphasized the need for continuity of care, honest communication, and the immediate disclosure of test results.

The stress caused by diagnostic uncertainty is in line with previous literature outside the neuro-oncology field.^3^ In neuro-oncology, uncertainty has predominantly been examined in terms of prognostic uncertainty, given the inevitability of disease recurrence, affecting both patients and caregivers.^4^^,^^23–28^ Other forms of uncertainty have also been reported, for example in relation to supportive therapies,^4^ or in anticipation of how the diagnosis and treatment side effects may ultimately influence quality of life.^28^ The short-term uncertainty and accompanying peak stress experienced while waiting for diagnostic scan results, as observed in our study, have also been reported in earlier research.^4^^,^^23^ However, the abovementioned studies have not explicitly examined the diagnostic uncertainty that patients must cope with when MRI results are themselves inconclusive.^4^^,^^23–28^ Our study identified a varying array of coping strategies in times of this diagnostic uncertainty, both among patients and between patients and their loved ones. This is consistent with previous literature on coping mechanisms throughout the glioma trajectory.^29^^,^^30^ Our observed variability in need for (diagnostic) information may be partly explained by the observed and previously reported^29^^,^^30^ variation in coping styles (eg, less need for diagnostic information in patients with an avoidant coping strategy), as well as by different phases of the treatment process.

From patients’ perspective, obtaining certainty faster was seen as the primary reason why the implementation of new diagnostic tests is considered valuable, with the ultimate aim of preserving quality of life, extending survival, or a combination of both. Importantly, the need for faster certainty is driven not only by the desire for medical actionability—informing treatment decisions and potentially improving survival or quality of life—but also by a sense of personal actionability, especially in situations without therapeutic consequences or options. In these cases, diagnostic clarity could stimulate patients to live more consciously and allow them to better plan their remaining years or months of life, also with the ultimate goal of preserving quality of life. This study illustrates how exploring patients’ perspectives introduces the goal of personal actionability of certain pieces of (diagnostic) information, on which they—including their loved ones—can act on, which challenges the common medical view that diagnostic tests should (always) have direct consequences for clinical management and treatment (ie, medical actionability). The main and common underlying goal—reserving quality of life—is a well-established central theme for glioma patients in the literature.^28^^,^^31^^,^^32^

Regarding the interaction with healthcare professionals in times of diagnostic uncertainty, all patients wished to participate in decision-making regarding the use of new diagnostic tests. Yet, the specific preferred role in the decision-making process varied, ranging from taking the lead to truly sharing the decision. Patient involvement in decisions such as choosing diagnostic tests was associated with a stronger sense of control over an otherwise uncertain situation. This desire for active involvement and the associated feeling of control has been highlighted in earlier studies of glioma patients^31^^,^^33^ and non-glioma patients.^32^^,^^34^

The preference for continuity in care members, honest communication, and the rapid disclosure of results and findings are consistent with previous studies.^4^^,^^31^^,^^35^^,^^36^ The wish for low-threshold access to professionals for questions found in our study has similarly been highlighted in prior work.^4^^,^^35^ These findings together reinforce the importance of a central, medically knowledgeable point of contact throughout the disease trajectory, such as a specialized nurse practitioner.

In line with our study, previous studies also show that patients want to be well informed but that the amount of desired information varies substantially across individuals.^4^^,^^28^^,^^31^^,^^33^ Our findings emphasize the importance of tailoring the information during a consultation to the patients’ informational needs.

This study has several strengths. It provides novel patient perspectives on the implementation of new diagnostic tests in neuro-oncology. It offers an in-depth focus on diagnostic uncertainty in glioma patients, while previous literature mainly concentrates on prognostic uncertainty.^4^^,^^23–28^ Moreover, the findings provide insights that are directly relevant to clinical practice while also informing the design of future research on diagnostic tests and their implementation. The burden of diagnostic uncertainty may be recognizable for experienced clinicians, and may strengthen clinicians’ efforts to prioritize rapid diagnostic testing, whenever feasible. The study team included experts from psychology, neurology, and communication, which may have provided more nuanced insights into the findings than if the study had been conducted solely from a neuro-oncology perspective.

At the same time, certain limitations should be acknowledged. The sample consisted of ten patients, all Dutch without a migration background. While some identified as Christian, no other religious or cultural perspectives were represented. Therefore, we cannot be certain that all perspectives were captured, although data saturation appeared to be sufficient. Our findings may not generalize to patients with different cultural or religious backgrounds. Nevertheless, variation in age, diagnosis, education level, and gender contributed to a diverse sample. Recruitment via a patient advisory panel may have introduced selection bias toward highly motivated participants, and the use of self-reported data carries a risk of recall bias. This possible motivation-based selection bias may have resulted in an overestimation of patients’ need for information and participation in diagnostic decision-making. The slight overrepresentation of lower-grade gliomas versus glioblastomas—when compared to the general glioma population—reflects the longer experience that lower-grade glioma patients have with follow-up MRI and diagnostic uncertainty; the needs of patients with a shorter prognosis—with glioblastoma but also brain metastases—may differ. Altogether, including the perspective of other religious, ethnic, and diagnostic backgrounds could add to the complete spectrum of patients’ perspectives and needs on this subject. This could be achieved by follow-up studies with an in-depth, qualitative design such as this one. Alternatively, adding questionnaires on this subject in cohort studies and trials in neuro-oncology could allow for a more systematic, quantitative analysis of the needs of the full spectrum of patients. Such a quantitative analysis also allows for the identification of relationships between patient characteristics and their diagnostic needs.

The study has implications for future research. Diagnostic tests that provide faster diagnostic certainty, even in the absence of therapeutic consequences, are important for patient actionability and its impact on quality of life. Future diagnostic trials could place greater emphasis on this fundamental aspect and consider in their implementation that a test can be valuable to patients even when it does not directly influence treatment decisions while remaining attentive to healthcare costs.

An ongoing diagnostic clinical trial using [^18^F]-fluoroethyltyrosine (FET)-PET/CT to distinguish treatment-associated changes from tumor progression focuses on this fundamental aspect within the context of cost-effectiveness.^37^ With respect to patients’ wishes for honest communication, rapid disclosure of test results, and continuity of care, future studies could further investigate these aspects to develop best practice guidelines and tools that promote high-quality communication in clinical practice.

In conclusion, this qualitative interview study explored glioma patients’ perspectives on diagnostic uncertainty related to treatment-associated changes and tumor progression, as well as on the implementation of new tests designed to reduce this uncertainty. Emphasizing patients’ perspective and quality of life in future quantitative diagnostic test research—even when tests do not directly influence treatment decisions—may help to better align implementation of these tests with what truly matters to patients, ultimately improving neuro-oncological care.

## Data Availability

Pseudonymized data supporting the findings of this study are available upon reasonable request from the corresponding author.
